# In-hospital mortality associated with transcatheter arterial embolization for treatment of hepatocellular carcinoma in patients on hemodialysis for end stage renal disease: a matched-pair cohort study using a nationwide database

**DOI:** 10.1259/bjro.20190004

**Published:** 2019-06-12

**Authors:** Masaya Sato, Ryosuke Tateishi, Hideo Yasunaga, Hiroki Matsui, Kiyohide Fushimi, Hitoshi Ikeda, Yutaka Yatomi, Kazuhiko Koike

**Affiliations:** 1 Department of Clinical Laboratory Medicine, Graduate School of Medicine, The University of Tokyo, Tokyo, Japan; 2 Department of Gastroenterology, Graduate School of Medicine, The University of Tokyo, Tokyo, Japan; 3 Department of Clinical Epidemiology and Health Economics, School of Public Health, The University of Tokyo, Tokyo, Japan; 4 Department of Health Policy and Informatics, Tokyo Medical and Dental University Graduate School of Medicine, Tokyo, Japan

## Abstract

**Objectives:**

No previous study has evaluated the risks associated with transcatheter arterial chemoembolization (TACE) for hepatocellular carcinoma in patients on hemodialysis (HD) for end stage renal disease (ESRD), because invasive treatment is rarely performed for such patients. We used a nationwide database to investigate in-hospital mortality and complication rates following TACE in patients on HD for ESRD.

**Methods:**

Using the Japanese Diagnosis Procedure Combination database, we enrolled patients on HD for ESRD who underwent TACE for hepatocellular carcinoma. For each patient, we randomly selected up to four non-dialyzed patients using a matched-pair sampling method based on the patient’s age, sex, treatment hospital, and treatment year. In-hospital mortality and complication rates were compared between dialyzed and non-dialyzed patients following TACE.

**Results:**

We compared matched pairs of 1551 dialyzed and 5585 non-dialyzed patients. Although the complication rate did not differ between the dialyzed and non-dialyzed ESRD patients [5.7% *vs* 5.8%, respectively; odds ratio, 0.99; 95% confidence interval (0.79–1.23); *p* = 0.90], the in-hospital mortality rate was significantly higher in dialyzed ESRD patients than in non-dialyzed patients [2.2% *vs* 0.97%, respectively; odds ratio, 2.21; 95% confidence interval (1.44–3.40); *p* < 0.001]. Among the dialyzed patients, the mortality rate was not significantly associated with sex, age, Charlson comorbidity index, or hospital volume.

**Conclusions:**

The in-hospital mortality rate following TACE was 2.2 % and was significantly higher in dialyzed than in non-dialyzed ESRD patients. The indications for TACE in HD-dependent patients should be considered carefully with respect to the therapeutic benefits *v* *s* risks.

**Advances in knowledge:**

In hospital mortality rate following TACE in dialyzed patients was more than twice compared to non-dialyzed patients. Post-procedural complication following TAE in ESRD onHD patients was 5.7%, and did not differ from that in non dialyzed patients.

## Introduction

Hepatocellular carcinoma (HCC) is the fifth most frequently diagnosed cancer and the third most frequent cause of cancer-related death.^1^ Hepatectomy, radiofrequency ablation (RFA), or liver transplantation were considered as the treatment of choice for HCC.^2–6^ However, although surveillance programs have reduced the proportion of HCC cases detected at an advanced stage in certain populations,^7,8^ only 30–40% of patients with HCC are candidates for curative treatment such as hepatic resection, liver transplantation, or percutaneous radiofrequency ablation.^9^ Transcatheter arterial chemoembolization (TACE) has been widely used as a palliative treatment for such patients.^10^ TACE often improves long-term outcomes in patients with unresectable HCC and thus is considered to be an acceptably effective treatment for patients with large or multifocal HCC who do not meet the indications for curative treatment.^11,12^

The number of end stage renal disease (ESRD) in patients undergoing hemodialysis (HD) have recently increased, primarily because of the increase in kidney failure induced by diabetes.^13,14^ Patients with ESRD undergoing HD have a higher incidence of having hepatitis virus infection and subsequent development of HCC.^15–17^ However, since dialyzed patients are typically associated with coagulopathies and immunocompromised,^18–22^ curative treatment procedures for HCC is rarely performed in such patients because of concerns about hemorrhagic complications. TACE plays an important role in the treatment of HCC that is otherwise considered inoperable. However, the risk of TACE-related complications in such patients remains unclear.

The Diagnosis Procedure Combination (DPC) database is a case-mix inpatient database in Japan that contains administrative claim data and discharge data of secondary and tertiary care hospitals,^23–25^ representing approximately 50% of inpatient admissions to such hospitals. The aim of the current study was to investigate the risk of mortality associated with TACE in dialyzed ESRD patients with HCC in a large patient sample using the nationwide DPC database.

## Methods

### Data source

The DPC database includes diagnoses (recorded with Japanese text and the International Classification of Diseases and Related Health Problems, 10th revision [ICD-10] codes), patient’s age, sex, demographics. It also contains therapeutic procedures encoded by the original Japanese codes, length of stay, discharge status including in-hospital death, and total costs. All 82 academic hospitals in Japan are required to participate in the DPC database, but participation is optional for community hospitals. The number of cases in the database were 2.65, 2.82, 2.78, 3.30, and 6.96 million in 2007, 2008, 2009, 2010, and 2011, respectively, representing approximately half of all inpatient to secondary and tertiary care hospital in Japan.

The requirement for informed consent was waived for this study because of the anonymous nature of the data. Study approval was obtained from the Institutional Review Board of the University of Tokyo.

### Samples

We extracted inpatient data over a total of 21 months (July 1, 2010–March 31, 2012) was from the database. We identified all patients with a diagnosis of HCC (ICD-10 code C220) from 12 million inpatients included during this period. We enrolled patients who received TACE. We then extracted the patients with a disease name of renal failure (ICD-10 codes N19, N180, and N19) who underwent HD. Next, we excluded patients who underwent TACE prior to RFA for the control of HCC or following curative treatment for management of hemorrhagic complications. We also excluded patients who underwent TACE to control tumor bleeding during emergency hospitalization. Finally, we matched each dialyzed patient with up to four non-dialyzed patients who underwent TACE. Matched patients were selected from the same hospital based on broad age categories (≤60, 61–70, 71–80, and ≥81 years), sex, and treatment year. We excluded patients for whom we could not find matched non-dialyzed patients. Charlson comorbidity index (CCI) was used to adjust for multiple comorbidities. The CCI is a widely used and well-validated index of comorbid condition that predicts 1 year mortality after hospital discharge^26^ and is based on Quan’s algorithm.^27^ Hospital volume was expressed as the number of cases during the study period, and was initially evaluated as a continuous variable.

### End points

We analyzed the in-hospital mortality rate after TACE as the primary end point of the current study. The secondary end point was the occurrence of post-procedural complications during hospitalization. We used ICD-10 codes to identify the complications (Supplementary Table 1).

Supplemental Material

### Statistical analysis

Patient characteristics were analyzed in terms of sex, age, and CCI. We also analyzed the relationship between in-hospital mortality or complication rate and hospital volume. Hospital volume for each therapeutic procedure during the survey period was determined using the unique identifier for each hospital, and categorized into three (low, intermediate, and high) categories, such that the numbers of patients in each group were almost equal. Univariate associations between each factor and in-hospital mortality was assessed using the χ^2^ test or Fisher’s exact test, as appropriate. Multivariate logistic regression analyses were used to examine the associations between outcomes and each variable. Multivariate analyses were performed with simultaneous forced entry of all variables. Univariate logistic regression was also used to compare the outcomes between the matched-pair dialyzed and non-dialyzed patients from the same hospital in terms of age groups, sex, and treatment year. Generalized estimating equations were used to account for clustering within each set of matched patients (*i.e.,* one in the case group and one to four in the control group). All analyses were performed using SPSS software (ver. 23.0; IBM Corp., Armonk, NY, USA).

## Results

### Patient characteristics

During the reference period, 1551 patients with ESRD or regular HD and 5585 matched non-dialyzed patients who received TACE were extracted. 77 dialyzed patients were excluded due to inability of finding matched non-dialyzed patients.

The sex and age distributions did not differ significantly between the dialyzed and non-dialyzed patients because we extracted matched samples based on sex and age groups. The proportion of patient without any information of aetiology was higher in dialyzed ESRD patients (Table 1).

**Table 1.** Clinical characteristics of dialyzed and non-dialyzed ESRD patients

Variable	Number of cases		*p-*value
	Dialyzed ESRD patients *n* (%)	Non-dialyzed ESRD patients *n* (%)	
Sex			0.3
Male	1389 (89.6)	5054 (90.5)	
Female	162 (10.4)	531 (9.5)	
Age (years)^a^			0.61
≤70	814 (52.5)	2888 (51.7)	
≥71	737 (47.5)	2697 (48.3)	
Etiology of cirrhosis			<0.001
Hepatitis B virus	78 (5.0)	655 (11.7)	
Hepatitis C virus	565 (36.5)	2293 (41.1)	
Hepatitis *B* + C virus	2 (0.1)	31 (0.6)	
Alcohol	68 (4.4)	331 (5.9)	
Other or not provided	838 (54.0)	2275 (40.7)	

ESRD, end stage renal disease.

a The median age was 70 years in both group

### Procedural outcomes

**Figure 1.**
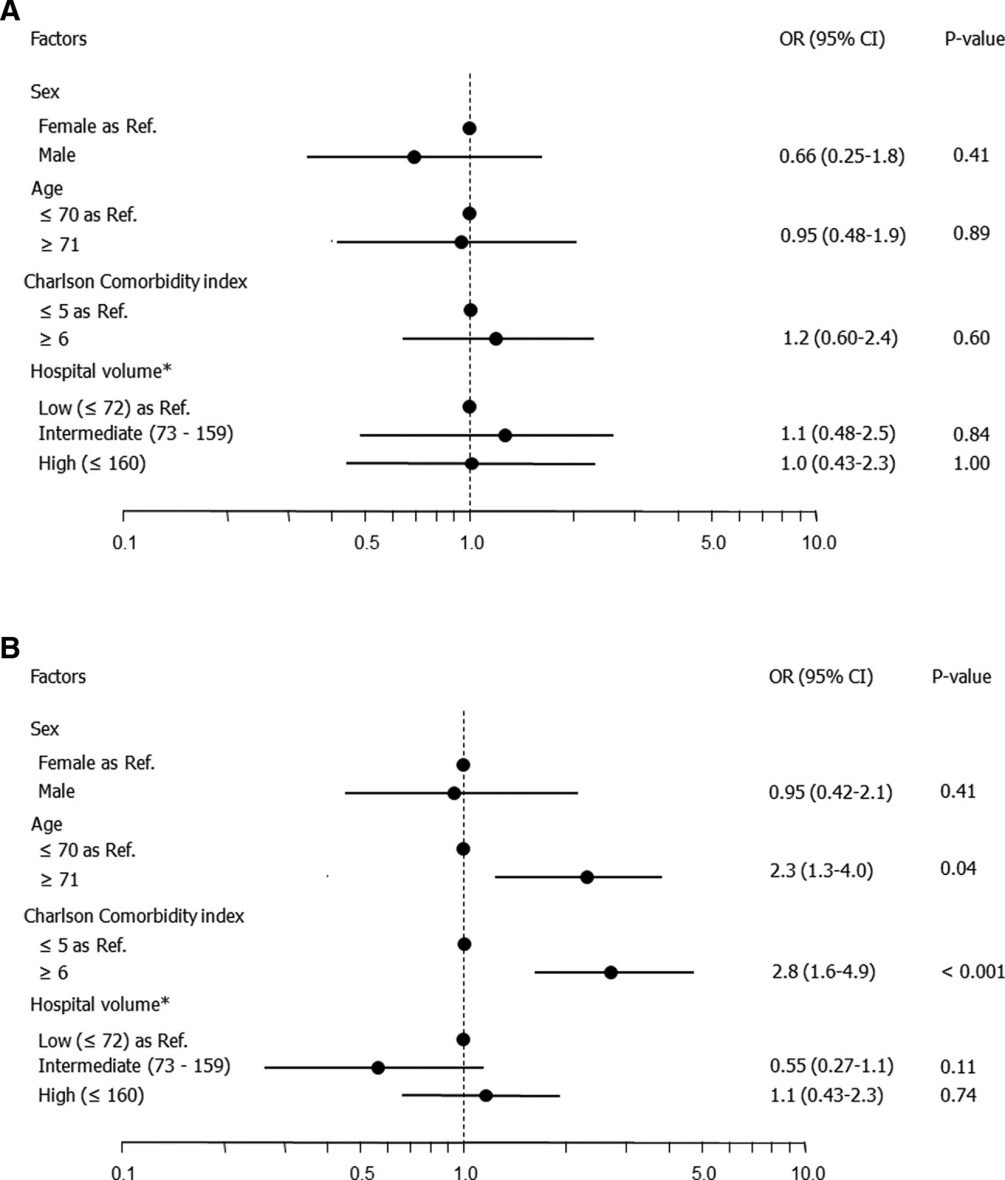
Multivariate logistic regression analysis of in-hospital mortality following TACE in (a) dialyzed and (b) non-dialyzed patients. TACE,transcatheter arterial chemoembolization.

In univariate analysis, the in-hospital mortality rates in dialyzed and non-dialyzed patients were 2.2% (34/1551) and 0.97% (56/5585), respectively (Table 2). Among the dialyzed patients, the mortality rate was not significantly associated with sex, age, CCI, or hospital volume. On the other hand, mortality was significantly higher among patients aged ≥71 years compared with those aged ≤70 years (*p* = 0.007) and among patients with a CCI ≥6 compared with those with a CCI ≤5 (*p* < 0.001) among non-dialyzed patients. Figure 1 shows multivariate associations between in-hospital mortality and patient characteristics in dialyzed and non-dialyzed patients. In multivariate analysis, no factor was significantly associated with in-hospital mortality among the dialyzed patients (Figure 1-a). Among the non-dialyzed patients, age ≥71 years (adjusted odds ratio [OR] 2.3, *p* = 0.04) and a CCI ≥6 (OR 2.8, *p* < 0.001) were significantly associated with a higher in-hospital mortality rate (Figure 1-b).

**Table 2.** Univariate analysis of risk factors for in-hospital death

Variable	Dialyzed ESRD patients			Non-dialyzed patients		
	*n*/N	% (95% CI)	*p*	*n*/N	% (95% CI)	*p*
Overall	34/1551	2.2 (1.5–3.0)		56/5585	0.97 (0.73–1.26)	
Sex			0.41			0.44
Female	5/162	3.1 (1.0–7.1)		7/531	1.3 (0.53–2.7)	
Male	29/1389	2.1 (1.4–3.0)		49/5054	0.97 (0.71–1.3)	
Age (years)			0.96			0.007
≤70	18/814	2.2 (1.3–3.5)		19/2888	0.66 (0.40–1.0)	
≥71	16/737	2.2 (1.2–3.5)		37/2697	1.4 (0.97–1.9)	
Charlson comorbidity index			0.61			<0.001
≤5	15/752	2.0 (1.1–3.3)		36/4593	0.78 (0.55–1.1)	
≥6	19/799	2.4 (1.4–3.7)		20/992	2.0 (1.2–3.1)	
Hospital volume^a^			0.97			0.16
High	11/518	2.1 (1.1–3.8)		23/1854	1.2 (0.79–1.9)	
Intermediate	12/517	2.3 (1.2–4.0)		11/1745	0.63 (0.32–1.1)	
Low	11/516	2.1 (1.1–3.8)		22/1986	1.1 (0.70–1.7)	

CI, confidence interval;ESRD, end stage renal disease; OR, odds ratio.

a Hospital volume was defined according to the number of cases per year. High, intermediate, and low hospital volumes represent hospitals with >159 cases, 73–159 cases, and <73 cases, respectively.

**Figure 2.**
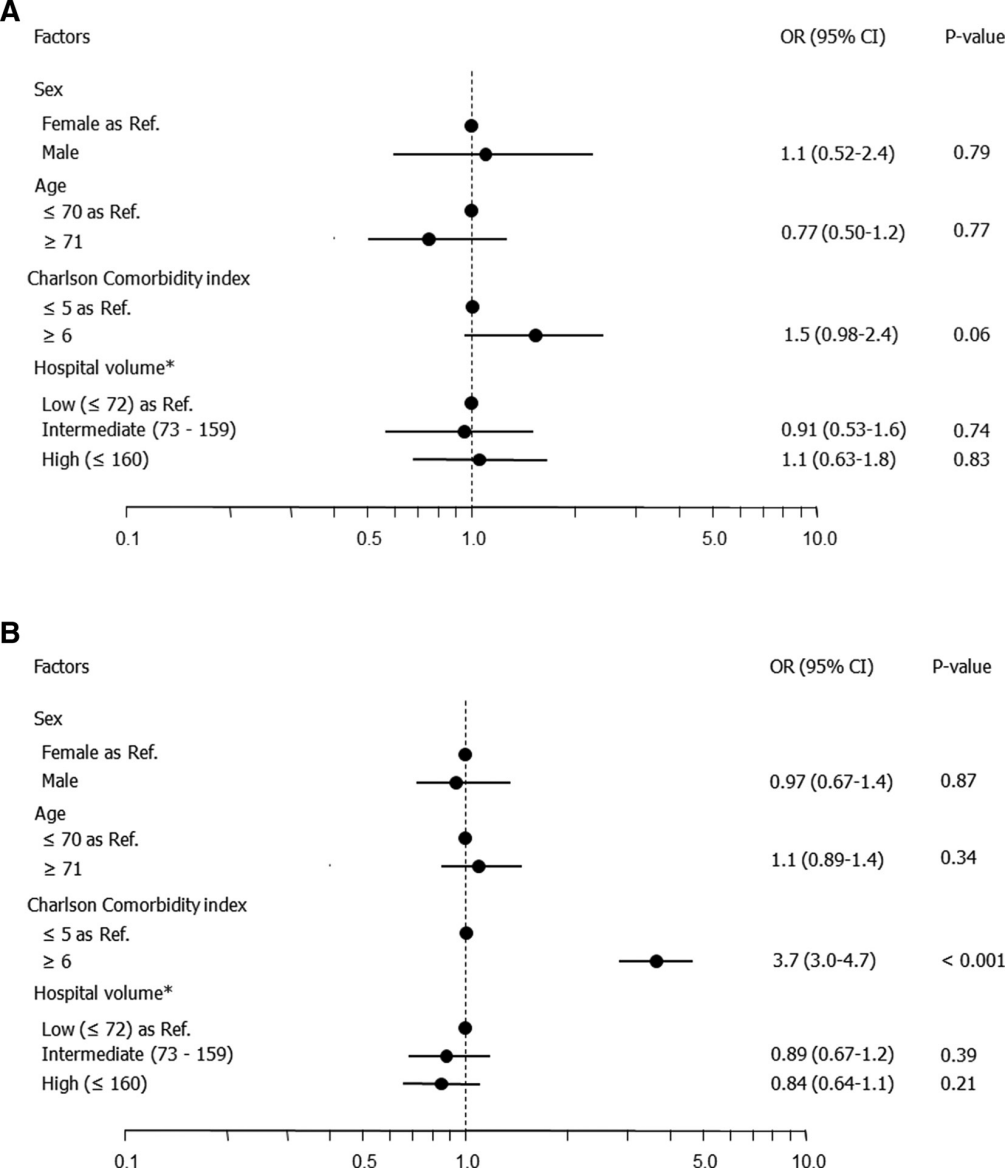
Multivariate logistic regression analysis of complication rates following TACE in (a) dialyzed and (b) non-dialyzed patients. TACE,transcatheter arterial chemoembolization.

Table 3 shows the complication rates and the univariate associations between patient characteristics and procedural backgrounds. The post-procedural complication rates in dialyzed and non-dialyzed patients were 5.7% (89/1551) and 5.8% (325/5585), respectively. Post-procedural complication rates were significantly higher among patients with a CCI ≥6 compared with those with a CCI ≤5 among both the dialyzed (*p* = 0.046) and non-dialyzed (*p* < 0.001) patients. In the multivariate analysis, patients with a CCI ≥6 remained significantly associated with a higher complication rate among the non-dialyzed patients only (OR 3.7, *p* < 0.001) (Figure 2).

**Table 3.** Univariate analysis of risk factors for complications

Variable	Dialyzed ESRD patients			Non-dialyzed patients		
	*n*/N	% (95% CI)	*p*	*n*/N	% (95% CI)	*p*
Overall	89/1551	5.7 (4.6–7.0)		325/5585	5.8 (5.2–6.5)	
Sex			0.64			0.42
Female	8/162	4.9 (2.2–9.5)		35/531	6.6 (4.6–9.0)	
Male	81/1389	5.8 (4.7–7.2)		290/5054	5.7 (5.1–6.4)	
Age (years)			0.17			0.90
≤70	53/814	6.5 (4.9–8.4)		167/2888	5.8 (5.0–6.7)	
≥71	36/737	4.9 (3.4–6.7)		158/2697	5.9 (5.0–6.8)	
Charlson comorbidity index			0.046			<0.001
≤5	34/752	4.5 (3.2–6.3)		189/4593	4.1 (3.6–4.7)	
≥6	55/799	6.9 (5.2–8.7)		136/992	13.7 (11.6–16.1)	
Hospital volume^a^			0.80			0.48
High	32/518	6.2 (4.3–8.6)		100/1854	5.4 (4.4–6.5)	
Intermediate	27/517	5.2 (3.5–7.5)		100/1745	5.7 (4.7–6.9)	
Low	30/516	5.8 (4.0–8.2)		125/1986	6.3 (5.3–7.5)	

CI, confidence interval;ESRD, end stage renal disease; OR, odds ratio.

a Hospital volume was defined according to the number of cases per year. High, intermediate, and low hospital volumes represent hospitals with >159 cases, 73–159 cases, and <73 cases, respectively.

### In-hospital mortality and complications following TACE

Table 4 shows the comparison of in-hospital mortality and complication rates between dialyzed and non-dialyzed patients using univariate logistic regression. The in-hospital mortality rate was significantly higher among dialyzed patients than among non-dialyzed patients (2.2% *vs* 0.97%, respectively; OR 2.21, *p* < 0.001). However, complication rates did not differ significantly between patients on HD with ESRD and non-dialyzed patients (5.7% *vs* 5.8%, OR 0.99, *p* = 0.90).

**Table 4.** Univariate logistic regression analysis of in-hospital mortality and complications in dialyzed and non-dialyzed ESRD patients

	**% (*n*/N**)	**Odds ratio (95% CI**)	***p***
In-hospital mortality			<0.001
Dialyzed ESRD patients	2.2% (34/1551)	2.21 (1.44–3.40)	
Non-dialyzed patients	0.97% (56/5585)		
Hemorrhagic complications			0.90
Dialyzed ESRD patients	5.7% (89/1551)	0.99 (0.79–1.23)	
Non-dialyzed patients	5.8% (325/5585)		

ESRD, end stage renal disease.

## Discussion

The number of patients with ESRD undergoing HD has increased recently.^13^ Generally, HD dependence is associated with higher mortality rates following procedures.^28^ Patients with ESRD on HD were reported to be associated with increased risk of having viral hepatitis and also having subsequent HCC.^15–17^ Therefore, for ESRD patients on regular dialysis, management of HCC is important.

In the current study, the in-hospital mortality and complication rates following TACE were 2.2%, and 5.7%, respectively. To the best of our knowledge, this is the first report of the quantitative risks associated with TACE for HCC in dialyzed ESRD patients. Although the complication rates following TACE were not significantly different between dialyzed and non-dialyzed patients, the in-hospital mortality rate was significantly higher in patients on HD for ESRD. This may be due to impaired immune function often associated with ESRD on HD patients.^20^ Also, patients with ESRD frequently have other major comorbidities, such as cardiovascular disease or diabetes mellitus, which can diminish their tolerance to complications. Therefore, complications that are tolerable in non-dialyzed patients may be critical problems in ESRD patients on HD.

In the current study, sex, age, CCI, and hospital volume did not affect the in-hospital mortality rate in dialyzed ESRD patients. This may be because ESRD patients on HD are at high risk of in-hospital mortality following TACE, which attenuates the influence of the analyzed factors such as age, sex, or other comorbidities which comprise the elements of CCI. Also, the sample size (number of event was 34 in total) may be underpowered to detect the statistical difference. In the current study, we could not identify dialyzed patients at a higher risk of TACE-related mortality. A further study with a larger sample is needed to identify risk factors for in-hospital mortality following TACE in ESRD patients on HD.

We previously reported the in-hospital mortality rate following RFA in dialyzed ESRD patients.^29^ In that study, the OR of in-hospital mortality in ESRD patients on HD compared with non-dialyzed patients was 7.77, which is markedly higher than that in the current study. ESRD patients on HD commonly demonstrate coagulation dysfunction due to platelet dysfunction from uremic toxins.^18,19^ Decreased tolerance to bleeding may increase the in-hospital mortality rate following RFA. Compared with RFA, the difference in risk between dialyzed and non-dialyzed patients was smaller. Information on the increased risk related to HCC treatment such as RFA or TACE in ESRD patients on HD compared with non-dialyzed patients is useful for determining the indications of treatment for HCC or explaining the risk of treatment to dialyzed ESRD patients or their family.

The current study had several limitations. Some important clinical data which may affect the procedural risk (*e.g.* laboratory data, Child-Pugh score, and size or location of tumor) were lacking in the database. Second, since most of the participating hospitals covered by the DPC database were secondary and tertiary level of facilities, the study population may not reflect the current medical context. Third, the in-hospital deaths stored in this database may include some deaths unrelated to treatment, because immediate cause of death is not a required item in this database. Similarly, we used post-operative diseases coded by ICD-10 to identify complications. Therefore, complications reported in the current study may leave room for the possibility that they include some diseases unrelated to treatment. However, even though patients on dialysis have shorter survival time than those without ESRDs, in-hospital mortality in elective hospitalization would be minimal when no intervention is performed. Also, compared to treatment-related mortality evaluated by operators, in-hospital mortality in the DPC database is a more robust concept in terms of objectivity.

## Conclusions

In conclusion, the in-hospital mortality rate following TACE was 2.2% in dialyzed patients. The mortality rate in dialyzed patients was higher than that in non-dialyzed patients. We quantitatively evaluated the risks associated with TACE for HCC in dialyzed ESRD patients. Indications for TACE in dialyzed ESRD patients should be considered carefully in based on the therapeutic benefits and risks.

## Disclosure

The eight authors are justifiably credited with authorship, according to the authorship criteria. In detail: MS—conception, design, analysis and interpretation of data, drafting of the manuscript, final approval given; RT—analysis and interpretation of data, critical revision of manuscript, final approval given; HY—collection and assembly of data, analysis and interpretation of data, critical revision, final approval given; HM—analysis and interpretation of data, critical revision, final approval given; KF—collection and assembly of data, final approval given; HI—interpretation of data, critical revision of manuscript, final approval given; YY—interpretation of data, critical revision of manuscript, final approval given; KK—interpretation of data, critical revision of manuscript, final approval given.
