# Supplementary material for: In-hospital mortality associated with transcatheter arterial embolization for treatment of hepatocellular carcinoma in patients on hemodialysis for end stage renal disease: a matched-pair cohort study using a nationwide database
Source: BJR Open. 2019 Jun 12;1(1):20190004. doi: 10.1259/bjro.20190004 (PMC7592431; doi:10.1259/bjro.20190004)
Supplement: Supplemental Material [file bjro.20190004.suppl-01.docx]

# Supplementary Table 1. Complication list

| Disease names | ICD-10 codes |
| --- | --- |
| Hemorrhage | D62, K768, R571, R58, T794, T810, T811, J942, S2721, S361, S3611, S3610, D500, K661 |
| Pulmonary embolism | I269 |
| Liver failure | K720, K729 |
| Peritoneal abscess | K650, K658, K659 |
| Pneumonia | J129, J13, J14, J150, J151, J152, J153, J154, J155, J156, J157, J158, J159, J160, J180, J181, J182, J188, J189, J690 |
| Hepatic infarction | K763 |
| Bile duct stenosis | K830, K831 |
| Allergy | T782, T784, T887 |
